# Driving Time to the Nearest Percutaneous Coronary Intervention-Capable Hospital and the Risk of Case Fatality in Patients with Acute Myocardial Infarction in Beijing

**DOI:** 10.3390/ijerph20043166

**Published:** 2023-02-10

**Authors:** Jie Chang, Qiuju Deng, Piaopiao Hu, Zhao Yang, Moning Guo, Feng Lu, Yuwei Su, Jiayi Sun, Yue Qi, Ying Long, Jing Liu

**Affiliations:** 1 Center for Clinical and Epidemiologic Research, Beijing An Zhen Hospital, Capital Medical University, Beijing Institute of Heart, Lung and Blood Vessel Diseases, National Clinical Research Center of Cardiovascular Diseases, Beijing 100029, China; 2 Beijing Municipal Key Laboratory of Clinical Epidemiology, Beijing 100029, China; 3 Beijing Municipal Health Big Data and Policy Research Center, Beijing Institute of Hospital Management, Beijing 100034, China; 4 School of Urban Design, Wuhan University, Wuhan 430072, China; 5 School of Architecture and Hang Lung Center for Real Estate, Key Laboratory of Eco Planning & Green Building, Ministry of Education, Tsinghua University, Beijing 100084, China

**Keywords:** acute myocardial infarction, case fatality, percutaneous coronary intervention, driving time

## Abstract

Timely arrival at a hospital capable of percutaneous coronary intervention (PCI) is critical in treating acute myocardial infarction (AMI). We examined the association between driving time to the nearest PCI-capable hospital and case fatality among AMI patients. A total of 142,474 AMI events during 2013–2019 from the Beijing Cardiovascular Disease Surveillance System were included in this cross-sectional study. The driving time from the residential address to the nearest PCI-capable hospital was calculated. Logistic regression was used to estimate the risk of AMI death associated with driving time. In 2019, 54.5% of patients lived within a 15-min drive to a PCI-capable hospital, with a higher proportion in urban than peri-urban areas (71.2% vs. 31.8%, *p* < 0.001). Compared with patients who had driving times ≤15 min, the adjusted odds ratios (95% CI, *p* value) for AMI fatality risk associated with driving times 16–30, 31–45, and >45 min were 1.068 (95% CI 1.033–1.104, *p* < 0.001), 1.189 (95% CI 1.127–1.255, *p* < 0.001), and 1.436 (95% CI 1.334–1.544, *p* < 0.001), respectively. Despite the high accessibility to PCI-capable hospitals for AMI patients in Beijing, inequality between urban and peri-urban areas exists. A longer driving time is associated with an elevated AMI fatality risk. These findings may help guide the allocation of health resources.

## 1. Introduction

Ischemic heart disease is a leading cause of death worldwide [1]. Acute myocardial infarction (AMI) is a serious, often-fatal manifestation of ischemic heart disease. Timely arrival to hospitals with percutaneous coronary intervention (PCI) capability after the onset of AMI is critical to lowering the risk of death [2,3]. It has been reported that 70.7% of patients hospitalized with AMI arrived at the hospital ≥2 h after symptom onset in China [4], while the obstacles for timely hospital arrival are yet to be elucidated. Geographic accessibility to hospitals has been thought to be associated with prolonged pre-hospital delay [5]. However, to what extent geographic accessibility may affect pre-hospital delay and prognosis for patients with AMI is poorly understood.

Several studies have investigated the association between geographic accessibility to PCI-capable hospitals and death among patients with AMI at the patient level or neighborhood level [6,7,8,9,10]. Three studies conducted at the patient level have assessed the association of straight-line distance and driving time to PCI-capable hospitals with the risk of in-hospital death [6,7,8]. However, half of all AMI deaths occur out of the hospital [11], and patients affected most by geographic accessibility may be more likely to die before reaching a PCI-capable hospital. Two published studies focused on AMI mortality rates (including both pre-hospital death and in-hospital death) at the neighborhood level but not the patient level [9,10]. Additionally, existing studies have failed to consider the impact of traffic conditions on driving time [8,10] or the impact of road networks on travel distance [6,7,9]. Therefore, the association between driving time to the nearest PCI-capable hospital and the risk of AMI death at the patient level is yet to be elucidated. Such investigations could guide the emergency care of AMI and affect decision-making on health resource allocations.

Using AMI events extracted from the Beijing Cardiovascular Disease Surveillance System (BCDSS), we aimed to quantify the driving time from the residential address to the nearest PCI-capable hospital considering traffic congestion during 2013–2019 in Beijing, and to further assess its association with the risk of AMI case fatality including both pre-hospital and in-hospital fatality.

## 2. Materials and Methods

### 2.1. Data Sources

Beijing, China’s capital city, comprises six districts as urban areas and ten districts as peri-urban areas [12]. We identified patients with AMI in Beijing using the BCDSS, which links routinely collected records in the Beijing Hospital Discharge Information System and the Beijing Vital Registration Monitoring System using personal identification information (Figure S1) [13]. The Beijing Hospital Discharge Information System covers admissions to all secondary- and tertiary-level hospitals in Beijing that are authorized to admit patients and provide comprehensive in-hospital medical, prevention, rehabilitation, and health care services. AMI admissions were identified on the basis of principal discharge diagnoses with International Classification of Diseases, Tenth Revision (ICD-10) codes I21–I22 (acute myocardial infarction and subsequent myocardial infarction) [14]. The Beijing Vital Registration Monitoring System covers all deaths in Beijing [15]. Deaths from AMI including both pre-hospital and in-hospital deaths were identified on the basis of the underlying cause of death using ICD-10 codes I21–I22 [14]. The diagnosis of AMI in the BCDSS has been validated as described in Supplementary Method S1. The study was approved by the ethics review committee at Beijing An Zhen Hospital, Capital Medical University, with a waiver of informed consent (2021139X).

### 2.2. Study Population

The system identified 179,866 AMI records among the permanent Beijing residents aged ≥35 years between 2013 and 2019. We took multiple steps to avoid double-counting AMI events. Patients who were discharged and then readmitted (including transferred patients) or died on the same day were deemed as single continuous care episodes (*n* = 5169). Patients discharged alive with a total length of stay of ≤1 day and without readmission or death on the same day were excluded (*n* = 2428) because these were unlikely to be AMI cases [16]. For patients with multiple events during the study period, we included a randomly selected single event and excluded other events for the same patient (*n* = 17,093) [17]. Information on the residential address for each patient was obtained from the BCDSS. To protect patient privacy, the number of the smallest unit for the address such as the apartment number was masked in the database. Patient addresses were geocoded to latitude and longitude coordinates, of which the 12,700 addresses that could not be geocoded to the patient level were excluded. After excluding patients with missing demographic information (*n* = 2), 142,474 AMI events were included in this analysis (Figure S2).

### 2.3. Study Outcomes

The primary outcome was AMI death and secondary outcomes were pre-hospital and in-hospital AMI deaths. AMI deaths were identified on the basis of the underlying cause of death on the death certificate. We defined hospitalized patients with a primary diagnosis of AMI and died during hospitalization as in-hospital AMI death and the remainder as pre-hospital AMI death.

### 2.4. Driving Time to the Nearest PCI-Capable Hospital

Hospitals in the BCDSS that reported performing PCI in patients were considered PCI-capable hospitals [18]. The addresses of PCI-capable hospitals were geocoded to the latitude and longitude coordinates. The road network system in Beijing has basically been stable in recent years. We considered the road network in 2021 and the impact of traffic conditions such as road congestion on driving time in a real setting [19]. We identified the nearest PCI-capable hospital for patients by computing the vehicle driving time along the road network between each patient–hospital pair using a web mapping application program interface [20] on the basis of the pair’s geographical coordinates. Because AMI most frequently occurs during the morning [21], we estimated the driving time during the morning peak traffic hours in 2021 and used the driving time during the evening peak traffic hours as a sensitivity analysis. Then, the driving time was adjusted using the Beijing Transport Development Annual Report to obtain the PCI-capable hospital accessibility in the year of the patient’s onset [22].

### 2.5. Covariates

Age, sex, marital status, comorbidities, day of the week (weekday or weekend), and year of the event were obtained from the BCDSS. Comorbidities were defined as any concomitant diagnosis including heart failure (ICD-10 code I50) and stroke (ICD-10 codes I60, I61, I63, and I64). The average year of education at the township level was extracted from the 2010 national population census [23]. Socioeconomic status included average per-capita disposable income at the district level from 2015 to 2019 derived from the statistical yearbook [24] and the proportion of unemployed derived at the district level from the 2010 national population census [23]. Cardiovascular risk factors at the district level included the average prevalence of hypertension, diabetes, hypercholesterolemia, and smoking in 2014 and 2017.

### 2.6. Statistical Analysis

Continuous variables were presented as means and standard deviations (SD), and differences between groups were compared using the one-way analysis of variance. Categorical variables were reported as numbers (percentages) and compared using the chi-squared test.

A multilevel logistic regression model was used to evaluate the risk of AMI death associated with driving time, which allowed the patients (level-1) to be nested within districts (level-2). The odds ratio (OR) and corresponding 95% confidence intervals (CI) were calculated. We stratified the driving time into four categories (≤15 min, 16–30 min, 31–45 min, and >45 min). Model 1 was univariate. Model 2 was adjusted for age and sex. Model 3 was further adjusted for marital status, years of education, day of the week, year of the event, per-capita disposable income (district level), and the proportion of unemployed (district level). Model 4 was additionally adjusted for heart failure, stroke, and the prevalence rate (district level) of hypertension, diabetes, hypercholesterolemia, and smoking. To assess possible effect modification, we conducted analyses stratified by age group (<65 and ≥65 years) and sex. Product terms between the driving time categories and dichotomized age and sex were additionally included in model 4, and a Wald test was used to calculate the *p* value for multiplicative interactions.

We further explored the association between driving time and the risk of pre-hospital and in-hospital death. In the analysis of the association between driving time and in-hospital death, patients who died pre-hospital were excluded. To adjust for potential selection bias in excluding pre-hospital deaths, we applied the inverse probability of selection weights (i.e., proportional to the reciprocal probability of having died before hospital admission) in a weighted regression in which an individual was assigned their selection weight [25]. Restricted cubic splines were used to flexibly model the concentration–response association between the driving time and the risk of pre-hospital death and in-hospital death. The number of knots was selected using the Akaike information criterion.

Three sensitivity analyses were performed to check the robustness of the results. First, we included the first event or the last event instead of a randomly selected single event for patients who had multiple AMI events during the study period. Second, we used the driving time during the evening peak traffic hours to assess the risk of AMI death associated with driving time. Third, we determined the driving distance along the road network from the patients’ residential address to the nearest PCI-capable hospital using the web mapping application program interface [20]. We then applied the driving distance as an alternative accessibility measurement to assess the association with AMI death.

All statistical analyses were performed using Stata software, version 17 (StataCorp., College Station, TX, USA). A 2-sided *p* < 0.05 was considered statistically significant.

## 3. Results

### 3.1. Characteristics of the Study Population

A total of 142,474 AMI events in Beijing between 2013 and 2019 were identified. The mean (SD) age was 69.3 (13.6) years, and 65.1% were male. Overall, 81.9% of patients were married, and 55.0% lived in urban areas. The numbers and proportions of patients within the driving time categories of ≤15, 16–30, 31–45, and >45 min were 67,821 (47.6%), 49,688 (34.9%), 18,038 (12.7%), and 6927 (4.8%), respectively. Patients with driving times ≤15 min were more likely to be male and had a higher proportion of heart failure and stroke. In addition, these patients were more likely to live in urban areas and in districts with higher per-capita disposable income and a lower proportion of unemployment (Table 1).

### 3.2. Driving Time to the Nearest PCI-Capable Hospital

**Figure 1:**
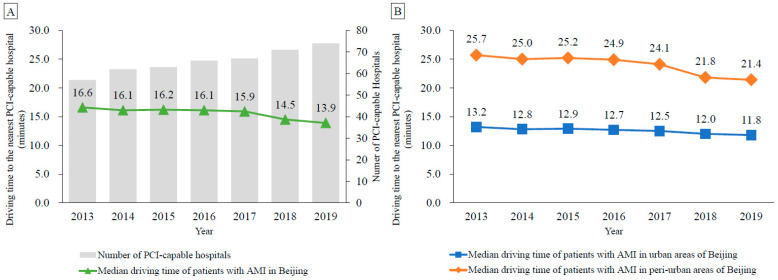
Median driving time to the nearest percutaneous coronary intervention-capable hospital in Beijing among patients with acute myocardial infarction, 2013–2019. (**A**) Median driving time of all patients in Beijing; (**B**) Median driving time of patients in urban and peri-urban areas of Beijing. Abbreviations: AMI, acute myocardial infarction; PCI, percutaneous coronary intervention.

During the study period, the median driving time to the nearest PCI-capable hospital for patients decreased gradually in Beijing from 16.6 to 13.9 min (Figure 1). In 2019, 54.5% (11,010/20,192) and 86.5% (17,471/20,192) of patients lived within a 15-min and 30-min drive to a PCI-capable hospital, respectively. Stratified analyses showed that the median driving time for urban patients decreased from 13.2 to 11.8 min over the study period, while that for peri-urban patients decreased from 25.7 to 21.4 min (Figure 1). In 2019, 71.2% (8284/11,628) and 99.1% (11,528/11,628) of patients in urban areas lived within a 15-min and 30-min drive to a PCI-capable hospital, respectively. In comparison, 31.8% (2726/8564) and 69.4% (5943/8564) of patients in peri-urban areas lived within a 15-min and 30-min drive to a PCI-capable hospital, respectively. In 2019, the proportion of patients living within a 15-min and 30-min drive to a PCI-capable hospital in urban areas was significantly higher than that in peri-urban areas (both *p* < 0.001).

### 3.3. Association between Driving Time and the Risk of AMI Fatality

**Figure 2:**
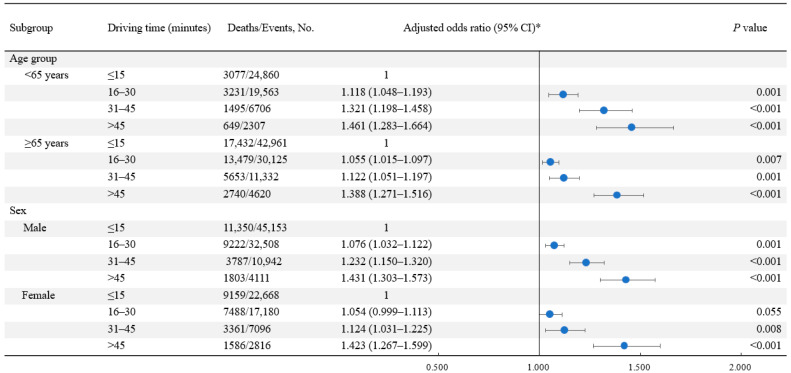
Subgroup analyses for the association between driving time to the nearest percutaneous coronary intervention-capable hospital and the risk of case fatality among patients with acute myocardial infarction. * Model adjusted for age, sex, marital status, day of the week, year of the event, heart failure, stroke, years of education, per-capita disposable income at the district level, proportion of unemployed at the district level, prevalence of hypertension at the district level, prevalence of diabetes at the district level, prevalence of hypercholesterolemia at the district level, and prevalence of smoking at the district level. Abbreviations: CI, confidence interval.

Among all AMI events, 33.5% (47,756/142,474) were fatal. The case fatality rates were 30.2% (20,509/67,821), 33.6% (16,710/49,688), 39.6% (7148/18,038), and 48.9% (3389/6927) among patients with driving times ≤15, 16–30, 31–45, and >45 min, respectively. Compared with patients who had driving times ≤15 min, the adjusted ORs (95% CI, *p* value) for the risk of AMI fatality among those with driving times of 16–30, 31–45, and >45 min were 1.068 (95% CI 1.033–1.104, *p* < 0.001), 1.189 (95% CI 1.127–1.255, *p* < 0.001), and 1.436 (95% CI 1.334–1.544, *p* < 0.001), respectively (Table 2). The association was stronger among younger patients (35–64 years) and male patients than among older patients (≥65 years) and female patients, respectively (both *P*_interaction_ < 0.001) (Figure 2).

### 3.4. Association between Driving Time and the Risk of Pre-Hospital and In-Hospital AMI Fatality

**Figure 3:**
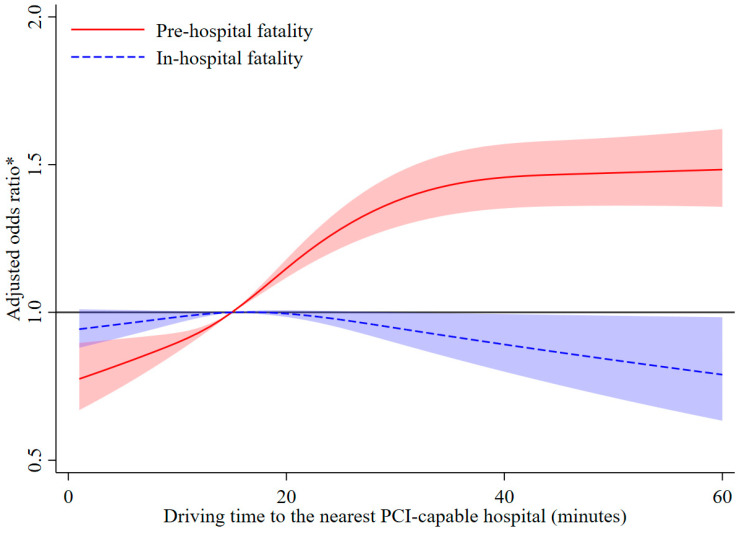
Restricted cubic spline analysis for the association between driving time to the nearest percutaneous coronary intervention-capable hospital and the risk of pre-hospital fatality and in-hospital fatality among patients with acute myocardial infarction. * Model adjusted for age, sex, marital status, day of the week, year of the event, heart failure, stroke, years of education, per-capita disposable income at the district level, proportion of unemployed at the district level, prevalence of hypertension at the district level, prevalence of diabetes at the district level, prevalence of hypercholesterolemia at the district level, and prevalence of smoking at the district level. In the analysis of the association between driving time and in-hospital death, the model additionally adjusted for potential selection bias.

A total of 21,312 pre-hospital fatal events and 26,444 in-hospital fatal events among patients with AMI during the study period were identified. Compared with patients who had driving times ≤15 min, those with driving times of 16–30 min (adjusted OR 1.243, 95% CI 1.174–1.316, *p* < 0.001), 31–45 min (adjusted OR 1.460, 95% CI 1.347–1.581, *p* < 0.001), and >45 min (adjusted OR 1.656, 95% CI 1.495–1.836, *p* < 0.001) had higher risks of pre-hospital fatality in the fully adjusted model. The adjusted ORs (95% CI, *p* value) for driving times of 16–30, 31–45, and >45 min associated with the risk of in-hospital fatality were 1.028 (0.979–1.080, *p* = 0.260), 0.851 (0.753–0.962, *p* = 0.010), and 0.833 (0.675–1.029, *p* = 0.091), respectively, adjusting for selection bias. Associations between driving time and the risks of both pre-hospital fatality and in-hospital fatality were nonlinear (*P*_nonlinearity_ < 0.001 for pre-hospital fatality; *P*_nonlinearity_ = 0.030 for in-hospital fatality). The risk of pre-hospital fatality increased with driving time and remained relatively flat when the driving time was more than approximately 40 min, while the risk of in-hospital fatality slightly decreased when the driving time was more than approximately 20 min (Figure 3).

### 3.5. Sensitivity Analyses

The results were similar to the main analyses when using the first event (Table S1) or the last event (Table S2) instead of a randomly selected single event for patients with multiple AMI events. The results did not change substantially when using the driving time during the evening peak traffic hours (Table S3) or the driving distance (Table S4) as surrogate measures of driving time during the morning peak traffic hours.

## 4. Discussion

Using city-wide data from Beijing, we found that the median driving time to the nearest PCI-capable hospital among patients with AMI decreased gradually from 2013 to 2019, and more than half of these patients lived within a 15-min drive to a PCI-capable hospital in 2019. However, a substantial disparity in driving time was found between patients living in urban areas and peri-urban areas. A longer driving time was associated with an increased risk of AMI fatality, especially pre-hospital AMI fatality. These findings reveal considerable inequality in the accessibility to PCI-capable hospitals within a city and highlight a need to improve the allocation of health resources for AMI acute treatment.

There is inconsistent evidence concerning the association of accessibility to PCI-capable hospitals with AMI mortality that includes both pre-hospital death and in-hospital death at the neighborhood level [9,10]. One study reported the adverse effects of a longer driving time to the nearest PCI-capable hospital on AMI mortality at the block group level in the United States [10]. However, another study conducted at the county level in the United States reported no significant association between distance to PCI-capable hospitals and AMI mortality, possibly because there is less variance at the larger scale (county level) than at the smaller scale (block group level) [9].

Our findings indicate the association between a longer driving time and an increased risk for AMI fatality at the patient level. A longer driving time to PCI-capable hospitals among patients with AMI is related to increased total ischemic time, which is associated with an increased risk of death [26]. Regarding the potential biological mechanism for this association, patients who have longer ischemic times may have less myocardium for salvage [26]. Previous studies conducted at the patient level have focused on the association between accessibility to PCI-capable hospitals and the risk of in-hospital death [6,7,8]. We further assessed the effect of driving time on the risk of AMI death stratified by pre-hospital and in-hospital death. Consistent with the results of prior studies [6,7,8], we did not find that increased driving time was associated with a higher risk of in-hospital death. A potential explanation is that patients with AMI who were most affected by accessibility might have died before reaching a PCI-capable hospital and were not included in those studies [6,7,8]. Therefore, it is likely that the increase in mortality risk associated with longer driving times may be underestimated among in-hospital deaths.

In our study, the association between driving time to the nearest PCI-capable hospital and the risk of AMI death differed by age and sex. The following reason may explain the stronger associations among younger patients than among older individuals. Compared with younger patients, older patients with AMI are more likely to use emergency medical services [27], which is associated with substantial reductions in ischemic time and treatment delay [27]. The discrepancy in associations between males and females may be because higher odds of not being aware of any AMI symptoms were associated with the male sex compared with the female sex [28].

Our study using city-wide data can help guide policy deliberations and the allocation of health resources. Although more than 70% of patients in urban areas lived within a 15-min drive to a PCI hospital in 2019, approximately 70% of peri-urban patients had a drive time of more than 15 min. Additionally, our previous work showed that rapid increases in AMI incidence were particularly evident in Beijing’s peri-urban areas [29]. These findings allow for the identification of within-city health care disparities to inform policy-making and cost-effective allocation of health resources. Our results also support Beijing’s recent health policy of providing high-quality medical resources in peri-urban areas [30], which may help reduce urban–rural inequality regarding access to health facilities and effectively reduce AMI deaths.

To our knowledge, this is the first study to estimate the driving time of patients with AMI to the nearest PCI-capable hospital in China using city-wide data and considering traffic congestion. Our system covered both hospitalized cases and pre-hospital deaths from AMI. Therefore, this is the most representative study providing data on the accessibility to PCI-capable hospitals in Beijing. Additionally, most previous studies have used straight-line distance [6,7,9] or speed limits to measure the driving time [10], which may lead to the underestimation of the driving time or distance, especially in megacities. In our study, the driving time based on the road network was estimated by taking the real traffic conditions into account, and the driving distance was estimated by considering the road network, thus providing a more accurate driving time and distance. Finally, this study included both pre-hospital and in-hospital deaths and comprehensively assessed the impact of driving time to the nearest PCI-capable hospital on the risk of death after the onset of AMI.

This study had several limitations. First, the driving time was calculated based on the residential address in our study, while the location where each patient experienced a heart attack was unavailable. However, the mean age of patients was 69.3 years in the current study. Previous research has reported that older adults spent 85% of their time at home [31], and a study conducted in China found that the daily activities of older adults were within a 15-min walking distance, especially a 5–10-min walking distance [32]. Therefore, we assumed that most AMI events occurred at or near the patients’ homes in our study. Second, the exact time of AMI onset was also unavailable. However, given that AMI onset is more common in the morning than at any other time of the day [21], we calculated the driving time during the morning peak traffic hours. The results in the sensitivity analyses when using the driving time during the evening peak traffic hours and driving distance as the accessibility indicators also support our main findings. Finally, whether patients were transported by ambulance was unavailable in our study. However, as found in our previous nationwide registry, only 11.6% of AMI patients in China were transported by ambulance [4]. Therefore, we estimated the driving time for non-ambulance transport. The pre-hospital survival rates could be higher due to the use of defibrillators and cardio-pulmonary resuscitation for AMI patients who reached the hospital by ambulance, although the proportion was low.

## 5. Conclusions

There is substantial disparity in accessibility to PCI-capable hospitals between urban and peri-urban areas in Beijing. A longer driving time to the nearest PCI-capable hospital is associated with a higher risk of AMI fatality. Considering the uneven distribution of PCI-capable hospital resources in Beijing, our study provides important information for further allocation of medical resources in megacities and may aid in improving the prognosis for AMI patients.

## Figures and Tables

**Table 1:** Characteristics of patients with acute myocardial infarction in Beijing between 2013 and 2019.

Characteristics	Total (*n* = 142,474)	Driving Time to the Nearest PCI-Capable Hospital (minutes)				*p* Value
		Driving Time: ≤15 (*n* = 67,821)	Driving Time: 16–30 (*n* = 49,688)	Driving Time: 31–45 (*n* = 18,038)	Driving Time: >45 (*n* = 6927)	
Patient-level characteristics						
Age, mean (SD), years	69.3 (13.6)	69.9 (13.7)	68.6 (13.7)	68.8 (13.4)	70.1 (13.3)	<0.001
Age groups, *n* (%)						<0.001
<65 years	53,436 (37.5)	24,860 (36.7)	19,563 (39.4)	6706 (37.2)	2307 (33.3)	
≥65 years	89,038 (62.5)	42,961 (63.3)	30,125 (60.6)	11,332 (62.8)	4620 (66.7)	
Male, *n* (%)	92,714 (65.1)	45,153 (66.6)	32,508 (65.4)	10,942 (60.7)	4111 (59.4)	<0.001
Marital status, *n* (%)						<0.001
Married	116,611 (81.9)	57,318 (84.5)	40,321 (81.2)	13,974 (77.5)	4998 (72.2)	
Not married	25,863 (18.1)	10,503 (15.5)	9367 (18.8)	4064 (22.5)	1929 (27.8)	
Comorbidity, *n* (%)						
Heart failure	46,068 (32.3)	24,057 (35.5)	15,531 (31.3)	4830 (26.8)	1650 (23.8)	<0.001
Stroke	8387 (5.9)	4603 (6.8)	2779 (5.6)	744 (4.1)	261 (3.8)	<0.001
Day of the week, *n* (%)						<0.001
Weekday	107,218 (75.3)	51,757 (76.3)	37,227 (74.9)	13,234 (73.4)	5000 (72.2)	
Weekend	35,256 (24.7)	16,064 (23.7)	12,461 (25.1)	4804 (26.6)	1927 (27.8)	
Region, *n* (%)						<0.001
Urban	78,394 (55.0)	50,871 (75.0)	26,061 (52.5)	1462 (8.1)	0	
Peri-urban	64,080 (45.0)	16,950 (25.0)	23,627 (47.5)	16,576 (91.9)	6927 (100.0)	
Years of education	11.3 (1.6)	12.1 (1.0)	11.2 (1.6)	9.5 (1.0)	8.7 (0.8)	<0.001
District-level characteristics						
Per capita disposable income, *n* (%)						<0.001
Quartile 1 (31,611–33,936)	21,454 (15.1)	4202 (6.2)	8515 (17.1)	7162 (39.7)	1575 (22.7)	
Quartile 2 (33,937–41,168)	32,769 (23.0)	8742 (12.9)	11,170 (22.5)	8108 (45.0)	4749 (68.6)	
Quartile 3 (41,169–65,966)	48,057 (33.7)	26,359 (38.9)	18,961 (38.2)	2134 (11.8)	603 (8.7)	
Quartile 4 (65,967–77,167)	40,194 (28.2)	28,518 (42.0)	11,042 (22.2)	634 (3.5)	0	
The proportion of unemployed, *n* (%)						<0.001
Quartile 1 (3.1–3.9)	37,020 (26.0)	17,975 (26.5)	12,723 (25.6)	3987 (22.1)	2335 (33.7)	
Quartile 2 (4.0–4.3)	44,359 (31.1)	26,292 (38.8)	15,028 (30.2)	2542 (14.1)	497 (7.2)	
Quartile 3 (4.4–4.7)	33,103 (23.2)	13,514 (19.9)	12,489 (25.1)	6324 (35.1)	776 (11.2)	
Quartile 4 (4.8–9.5)	27,992 (19.7)	10,040 (14.8)	9448 (19.1)	5185 (28.7)	3319 (47.9)	

Abbreviations: PCI, percutaneous coronary intervention; SD, standard deviation.

**Table 2:** Association between driving time to the nearest percutaneous coronary intervention-capable hospital and the risk of case fatality among patients with acute myocardial infarction.

Driving Time (minutes)	Deaths/Events, No.	Model 1 *		Model 2 †		Model 3 ‡		Model 4 §	
		OR (95% CI)	*p* Value	Adjusted OR (95% CI)	*p* Value	Adjusted OR (95% CI)	*p* Value	Adjusted OR (95% CI)	*p* Value
≤15	20,509/67,821	1		1		1		1	
16–30	16,710/49,688	1.210 (1.177–1.243)	<0.001	1.291 (1.253–1.329)	<0.001	1.071 (1.037–1.106)	<0.001	1.068 (1.033–1.104)	<0.001
31–45	7148/18,038	1.703 (1.635–1.774)	<0.001	1.759 (1.683–1.838)	<0.001	1.211 (1.149–1.277)	<0.001	1.189 (1.127–1.255)	<0.001
>45	3389/6927	2.566 (2.428–2.713)	<0.001	2.467 (2.322–2.620)	<0.001	1.501 (1.399–1.611)	<0.001	1.436 (1.334–1.544)	<0.001

* Model 1: Not adjusted; † Model 2: Adjusted for age and sex; ‡ Model 3: Model 2 + marital status + day of the week + year of the event + years of education + per-capita disposable income at the district level + the proportion of unemployed population at the district level; § Model 4: Model 3 + heart failure + stroke + the prevalence of hypertension at the district level + the prevalence of diabetes at the district level + the prevalence of hypercholesterolemia at the district level + the prevalence of smoking at the district level. Abbreviations: OR, odds ratio; CI, confidence interval.

## Data Availability

The data used for this study were obtained from the Beijing Municipal Health Big Data and Policy Research Center and cannot be shared publicly given the institutional regulations and the data confidentiality agreement. Similar data may be requested by researchers from the above data holder authorities for research purposes. The analytical methods can be reproduced based on the details provided in this article, and the statistical code is available upon request.

## Supplementary Materials

The following supporting information can be downloaded at: https://www.mdpi.com/article/10.3390/ijerph20043166/s1, Method S1: Validation of the diagnosis of acute myocardial infarction in the Beijing Cardiovascular Disease Surveillance System. Table S1: Association between driving time to the nearest percutaneous coronary intervention-capable hospital and the risk of case fatality among patients with the first acute myocardial infarction event, 2013–2019. Table S2: Association between driving time to the nearest percutaneous coronary intervention-capable hospital and the risk of case fatality among patients with the last acute myocardial infarction event, 2013–2019. Table S3: Association between driving time to the nearest percutaneous coronary intervention-capable hospital during evening peak traffic hours and the risk of case fatality among patients with acute myocardial infarction. Table S4: Association between driving distance to the nearest percutaneous coronary intervention-capable hospital and the risk of case fatality among patients with acute myocardial infarction. Figure S1: Data source and linkage of the Beijing Cardiovascular Disease Surveillance System. Figure S2: Flowchart of the study population. Reference [33] is cited in the Supplementary Materials.

## Abbreviations

PCI: percutaneous coronary intervention; AMI: acute myocardial infarction; BCDSS: Beijing Cardiovascular Disease Surveillance System; ICD-10: International Classification of Diseases, Tenth Revision; SD: standard deviation; OR: odds ratio; CI: confidence interval.
