# Supplementary material for: Driving Time to the Nearest Percutaneous Coronary Intervention-Capable Hospital and the Risk of Case Fatality in Patients with Acute Myocardial Infarction in Beijing
Source: Int J Environ Res Public Health. 2023 Feb 10;20(4):3166. doi: 10.3390/ijerph20043166 (PMC9961430; doi:10.3390/ijerph20043166)
Supplement: Supplementary file 1 [file ijerph-20-03166-s001.zip › ijerph-2080380-supplementary.pdf]

SUPPLEMENTAL MATERIAL

Driving Time to the Nearest Percutaneous Coronary Intervention-Capable Hospital and the Risk of Case Fatality in Patients With Acute Myocardial Infarction in Beijing

Method S1. Validation of the Diagnosis of Acute Myocardial Infarction in the Beijing Cardiovascular Disease Surveillance System

Table S1. Association Between Driving Time to the Nearest Percutaneous Coronary Intervention-Capable Hospital and the Risk of Case Fatality Among Patients With the First Acute Myocardial Infarction Event, 2013–2019

Table S2. Association Between Driving Time to the Nearest Percutaneous Coronary Intervention-Capable Hospital and the Risk of Case Fatality Among Patients With the Last Acute Myocardial Infarction Event, 2013–2019

Table S3. Association Between Driving Time to the Nearest Percutaneous Coronary Intervention-Capable Hospital During Evening Peak Traffic Hours and the Risk of Case Fatality Among Patients With Acute Myocardial Infarction

Table S4. Association Between Driving Distance to the Nearest Percutaneous Coronary Intervention-Capable Hospital and the Risk of Case Fatality Among Patients with Acute Myocardial Infarction

Figure S1. Data Source and Linkage of the Beijing Cardiovascular Disease Surveillance System

Figure S2. Flowchart of the Study Population

Supplemental Methods

Method S1. Validation of the Diagnosis of Acute Myocardial Infarction in the Beijing Cardiovascular Disease Surveillance System

Diagnoses in the Beijing Cardiovascular Disease Surveillance System (BCDSS) have been previously validated, with "almost perfect" or "substantial" agreement (kappa statistic range: 0.725 to 0.880) between International Classification of Diseases code-based diagnoses in the system and clinical diagnoses in the original hospital charts. The positive predictive values of acute myocardial infarction diagnosis in the BCDSS were 94.4% and 87.9% when compared with the criteria of the World Health Organization Monitoring Trends and Determinants in Cardiovascular Disease (WHO MONICA) project and the third universal definition of acute myocardial infarction, respectively. The corresponding negative predictive values were 96.1% and 97.0%, respectively [33].

Supplemental Tables

Table S1. Association Between Driving Time to the Nearest Percutaneous Coronary Intervention-Capable Hospital and the Risk of Case Fatality Among Patients With the First Acute Myocardial Infarction Event, 2013–2019

Driving time (minutes)	Deaths/Events, No.	Model 1*		Model 2†		Model 3‡		Model 4§	
		OR (95% CI)	*P* value	Adjusted OR (95% CI)	*P* value	Adjusted OR (95% CI)	*P* value	Adjusted OR (95% CI)	*P* value
≤15	19,498/67,695	1		1		1		1	
16–30	16,092/49,767	1.220 (1.187–1.254)	<0.001	1.298 (1.260–1.337)	<0.001	1.084 (1.050–1.120)	<0.001	1.079 (1.043–1.115)	<0.001
31–45	6924/18,051	1.718 (1.649–1.790)	<0.001	1.771 (1.693–1.851)	<0.001	1.231 (1.168–1.297)	<0.001	1.205 (1.142–1.273)	<0.001
>45	3304/6929	2.598 (2.457–2.747)	<0.001	2.488 (2.342–2.643)	<0.001	1.535 (1.430–1.647)	<0.001	1.465 (1.361–1.576)	<0.001

* Model 1: Not adjusted
† Model 2: Adjusted for age and sex
‡ Model 3: Model 2 + marital status + day of the week + year of the event + years of education + per-capita disposable income at the district level + the proportion of unemployed population at the district level
§ Model 4: Model 3 + heart failure + stroke + the prevalence of hypertension at the district level + the prevalence of diabetes at the district level + the prevalence of hypercholesterolemia at the district level + the prevalence of smoking at the district level
Abbreviations: OR, odds ratio; CI, confidence interval.

Table S2. Association Between Driving Time to the Nearest Percutaneous Coronary Intervention-Capable Hospital and the Risk of Case Fatality Among Patients With the Last Acute Myocardial Infarction Event, 2013–2019

Driving time (minutes)	Deaths/Events, No.	Model 1*		Model 2†		Model 3‡		Model 4§	
		OR (95% CI)	*P* value	Adjusted OR (95% CI)	*P* value	Adjusted OR (95% CI)	*P* value	Adjusted OR (95% CI)	*P* value
≤15	21,748/67,961	1		1		1		1	
16–30	17,483/49,608	1.203 (1.171–1.236)	<0.001	1.289 (1.252–1.327)	<0.001	1.069 (1.035–1.103)	<0.001	1.064 (1.029–1.099)	<0.001
31–45	7429/18,025	1.687 (1.620–1.757)	<0.001	1.752 (1.676–1.831)	<0.001	1.211 (1.149–1.276)	<0.001	1.185 (1.123–1.251)	<0.001
>45	3486/6912	2.536 (2.399–2.681)	<0.001	2.446 (2.303–2.599)	<0.001	1.491 (1.389–1.601)	<0.001	1.424 (1.324–1.532)	<0.001

* Model 1: Not adjusted
† Model 2: Adjusted for age and sex
‡ Model 3: Model 2 + marital status + day of the week + year of the event + years of education + per-capita disposable income at the district level + the proportion of unemployed population at the district level
§ Model 4: Model 3 + heart failure + stroke + the prevalence of hypertension at the district level + the prevalence of diabetes at the district level + the prevalence of hypercholesterolemia at the district level + the prevalence of smoking at the district level
Abbreviations: OR, odds ratio; CI, confidence interval.

Table S3. Association Between Driving Time to the Nearest Percutaneous Coronary Intervention-Capable Hospital During Evening Peak Traffic Hours and the Risk of Case Fatality Among Patients With Acute Myocardial Infarction

Driving time (minutes)	Deaths/Events, No.	Model 1*		Model 2†		Model 3‡		Model 4§	
		OR (95% CI)	*P* value	Adjusted OR (95% CI)	*P* value	Adjusted OR (95% CI)	*P* value	Adjusted OR (95% CI)	*P* value
≤15	20,477/68,003	1		1		1		1	
16–30	16,925/50,500	1.241 (1.208–1.275)	<0.001	1.308 (1.270–1.347)	<0.001	1.094 (1.060–1.130)	<0.001	1.097 (1.061–1.134)	<0.001
31–45	6732/16,474	1.833 (1.759–1.911)	<0.001	1.857 (1.775–1.943)	<0.001	1.281 (1.214–1.351)	<0.001	1.249 (1.182–1.320)	<0.001
>45	3622/7497	2.422 (2.296–2.556)	<0.001	2.332 (2.200–2.472)	<0.001	1.462 (1.365–1.566)	<0.001	1.405 (1.308–1.509)	<0.001

* Model 1: Not adjusted
† Model 2: Adjusted for age and sex
‡ Model 3: Model 2 + marital status + day of the week + year of the event + years of education + per-capita disposable income at the district level + the proportion of unemployed population at the district level
§ Model 4: Model 3 + heart failure + stroke + the prevalence of hypertension at the district level + the prevalence of diabetes at the district level + the prevalence of hypercholesterolemia at the district level + the prevalence of smoking at the district level
Abbreviations: OR, odds ratio; CI, confidence interval.

Table S4 Association Between Driving Distance to the Nearest Percutaneous Coronary Intervention-Capable Hospital and the Risk of Case Fatality Among Patients With Acute Myocardial Infarction

Driving Distance (kilometers)	Deaths/Events, No.	Model 1*		Model 2†		Model 3‡		Model 4§	
		OR (95% CI)	*P* value	Adjusted OR (95% CI)	*P* value	Adjusted OR (95% CI)	*P* value	Adjusted OR (95% CI)	*P* value
≤5	24,501/81,583	1		1		1		1	
6–10	9145/27,122	1.232 (1.194–1.271)	<0.001	1.311 (1.268–1.356)	<0.001	1.090 (1.050–1.132)	<0.001	1.080 (1.039–1.123)	<0.001
11–20	7466/19,146	1.609 (1.550–1.670)	<0.001	1.675 (1.608–1.747)	<0.001	1.253 (1.194–1.314)	<0.001	1.220 (1.161–1.281)	<0.001
>20	6644/14,623	2.266 (2.172–2.363)	<0.001	2.208 (2.109–2.311)	<0.001	1.462 (1.379–1.549)	<0.001	1.395 (1.314–1.482)	<0.001

* Model 1: Not adjusted
† Model 2: Adjusted for age and sex
‡ Model 3: Model 2 + marital status + day of the week + year of the event + years of education + per-capita disposable income at the district level + the proportion of unemployed population at the district level
§ Model 4: Model 3 + heart failure + stroke + the prevalence of hypertension at the district level + the prevalence of diabetes at the district level + the prevalence of hypercholesterolemia at the district level + the prevalence of smoking at the district level
Abbreviations: OR, odds ratio; CI, confidence interval.

Supplemental Figures

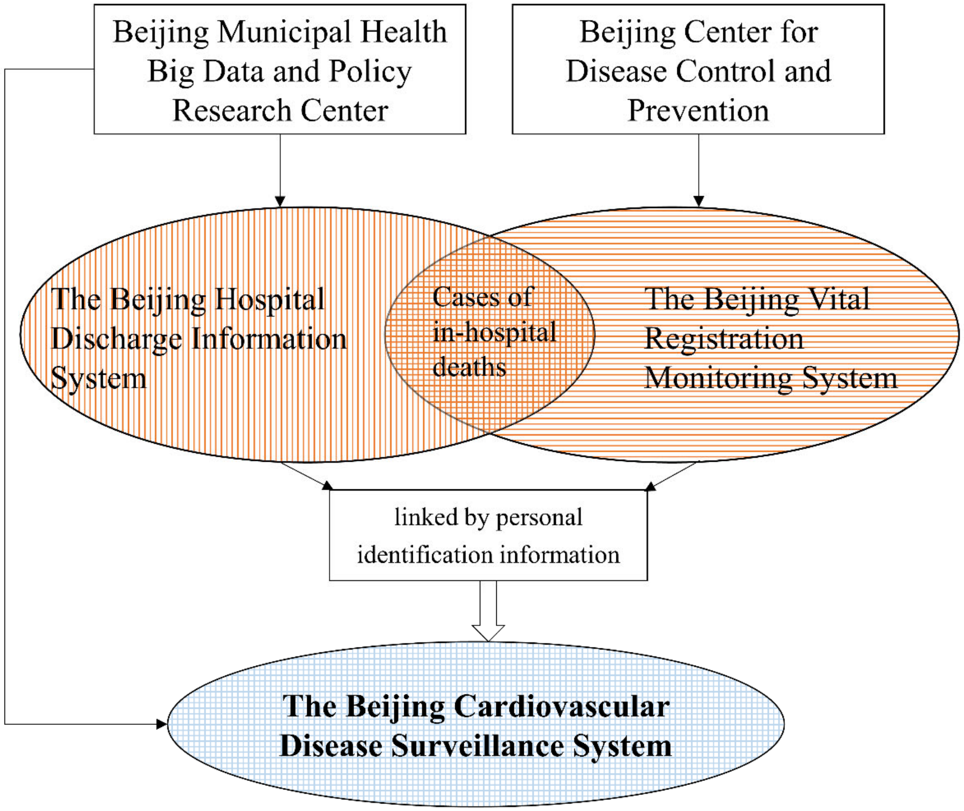

Figure S1. Data Source and Linkage of the Beijing Cardiovascular Disease Surveillance System

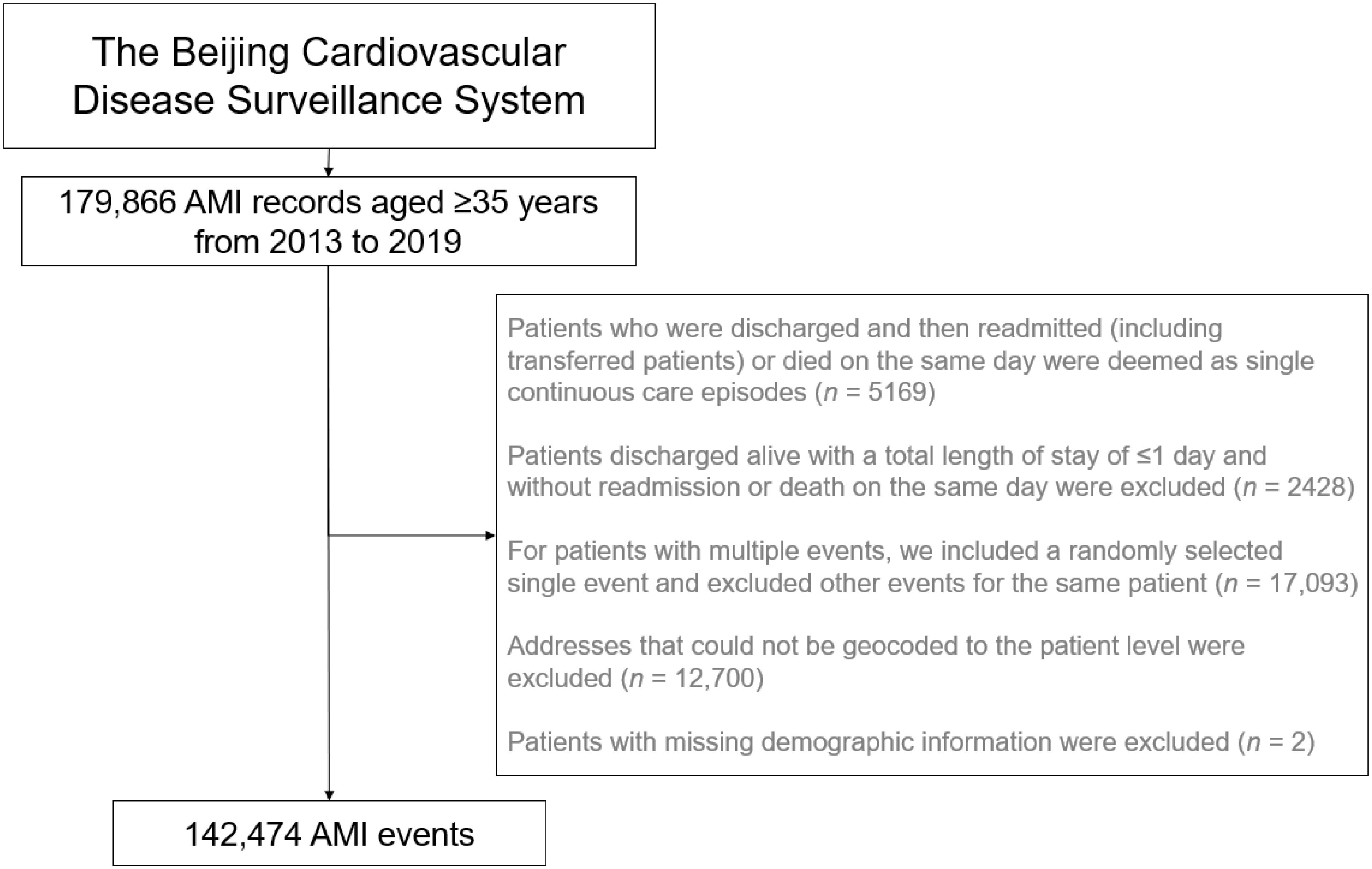

Figure S2. Flowchart of the Study Population

Abbreviations: AMI, acute myocardial infarction.

References

33. Zhang Q, Zhao D, Xie W, Xie X, Guo M, Wang M, et al. Recent trends in hospitalization for acute myocardial infarction in Beijing: increasing overall burden and a transition from ST-segment elevation to non-ST-segment elevation myocardial infarction in a population-based study. *Medicine (Baltimore)* 2016;95:e2677.
